# A Unique Presentation of Secondary Syphilis With Painful Target Lesions

**DOI:** 10.7759/cureus.58382

**Published:** 2024-04-16

**Authors:** Namratha Puttur, Shubham Deokar, Kshitiz Lakhey, Asharbh Raman

**Affiliations:** 1 Dermatology, Venereology and Leprosy, Dr. D. Y. Patil Medical College, Hospital & Research Centre, Dr. D. Y. Patil Vidyapeeth (Deemed to be University), Pune, IND

**Keywords:** infectious disease diagnosis, sexually transmitted infection (sti), hiv-syphilis co-infection, hiv aids, atypical syphilis

## Abstract

Syphilis, caused by *Treponema pallidum subsp. pallidum*, remains a global health challenge, with a significant burden of new cases annually. The disease disproportionately affects men who have sex with men (MSMs) and endemic, low-income regions. While secondary syphilis typically manifests with a polymorphic rash, individuals with human immunodeficiency virus (HIV) coinfection may present with varied signs and symptoms. Here, we report a case of a 21-year-old male student with painful target lesions on his genitalia, deviating from the typical syphilis presentation. He was found to have concurrent molluscum contagiosum and HIV-1 infection. Serologic testing confirmed syphilis and anti-HIV-1 antibodies. Prompt initiation of antiretroviral therapy and benzathine penicillin G led to symptom resolution. This case highlights the importance of recognizing atypical painful target lesions as a potential manifestation of syphilis, especially in patients with HIV coinfection, to ensure timely diagnosis and treatment.

## Introduction

Syphilis is a disease caused by the spirochete *Treponema (T.) pallidum* *subsp.* *pallidum*. It is a persistent global health concern with an annual incidence of 5 to 12 million cases despite effective antibiotic treatments [1,2]. The disease has a significant concentration of cases in endemic, low-income regions and among populations of men who have sex with men (MSMs). MSMs demonstrate a disproportionate susceptibility to syphilis compared to women, with 402.0 cases of primary and secondary syphilis per 100,000 men, in contrast to 10.8 cases per 100,000 females [3,4].

Patients having secondary syphilis usually present with a generalized polymorphic rash along with low-grade fever, lymphadenopathy, and hepatosplenomegaly. However, the presentation may differ in individuals co-infected with human immunodeficiency virus (HIV). Syphilis may progress more rapidly or present with varied signs and symptoms in people living with HIV (PLHIV) [5]. This interplay between syphilis and HIV adds another layer of complexity to the disease.

Notably, our case deviates from the typical clinical presentation with painful target lesions highlighting the diverse clinical spectrum of syphilis.

## Case presentation

A 21-year-old male student presented to the dermatology outpatient department with complaints of lesions on his scrotum and penis for the past month, which were accompanied by pain and slight watery discharge. He also had painful ulcers in his oral cavity for the last 20 days. The patient disclosed a history of engaging in both heterosexual and homosexual intercourse with occasional visits to commercial sex workers. However, he denied any previous history of sexually transmitted infections (STIs).

**Figure 1:**
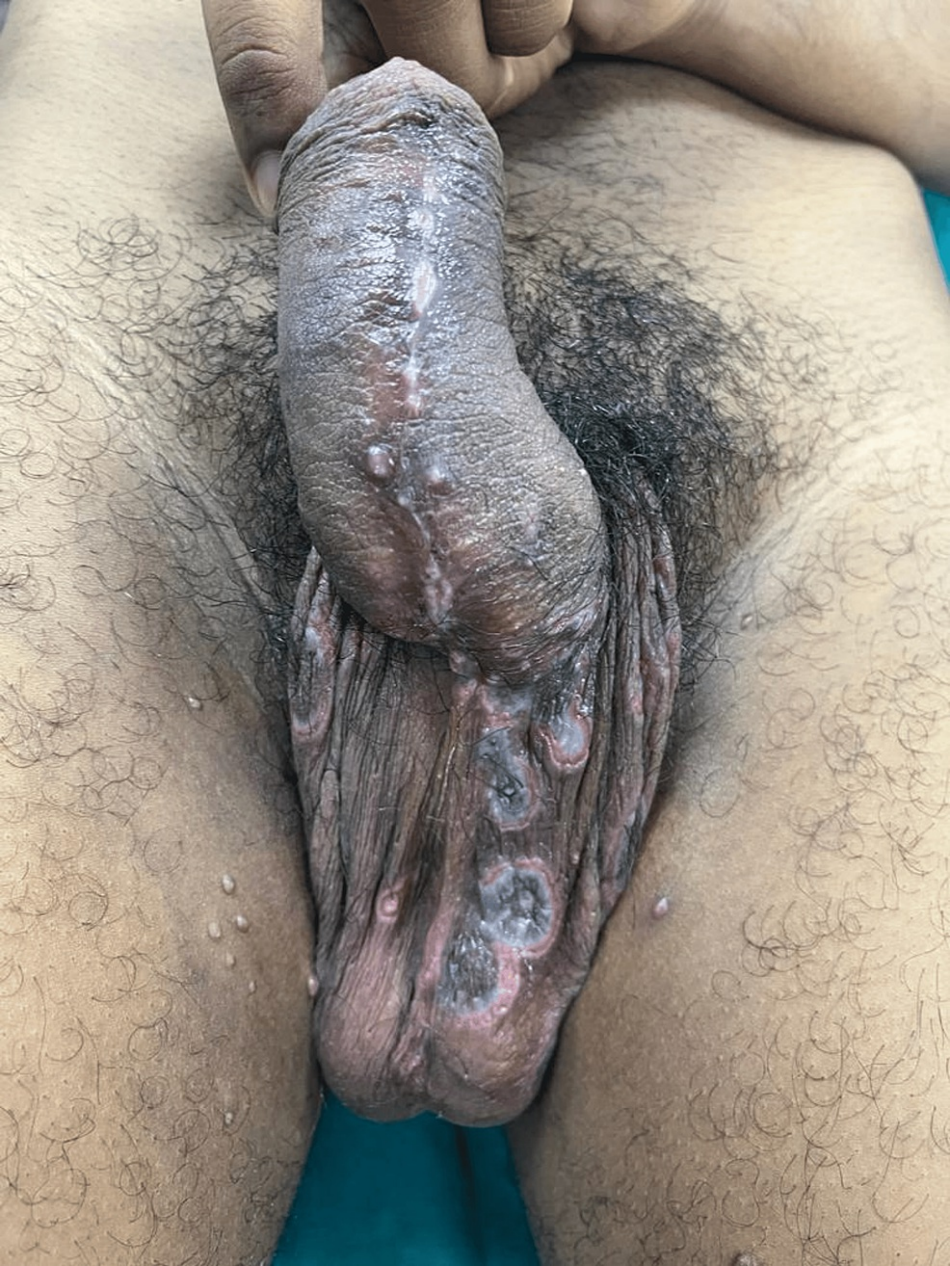
Atypical lesions of secondary syphilis and Molluscum contagiosum in an HIV-positive patient Multiple, atypical, target-shaped plaques with the central clearing surrounded by a moist white zone and an erythematous erosive border on the patient's penile shaft and scrotum, with multiple, dome-shaped, umbilicated, pearly white papules on the medial aspect of bilateral thighs

**Figure 2:**
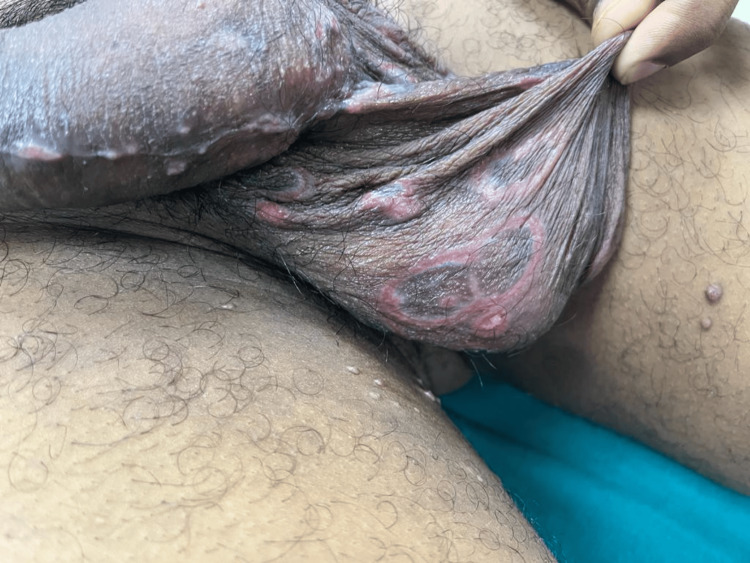
Atypical lesions of secondary syphilis and Molluscum contagiosum in an HIV-positive patient Multiple, atypical, target-shaped plaques of secondary syphilis on the patient's penile shaft and scrotum, with Molluscum contagiosum lesions over the medial aspect of bilateral thighs

**Figure 3:**
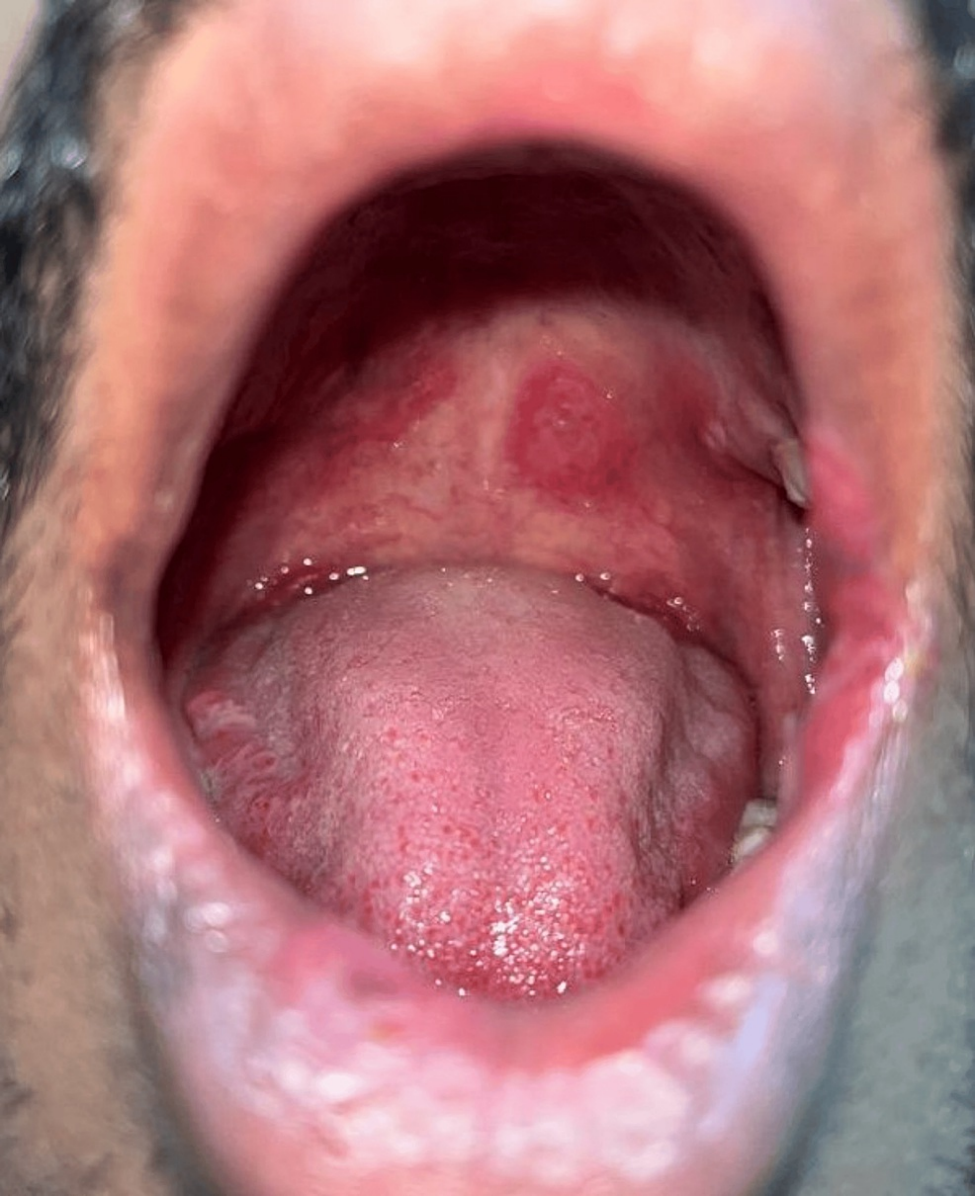
Oral lesions of secondary syphilis Multiple, tender, erythematous ulcers on the hard palate.

The objective examination revealed the presence of multiple, tender, target-shaped plaques with the central clearing surrounded by a moist white zone and an erythematous erosive border on the patient's penile shaft and scrotum. Multiple dome-shaped, umbilicated, pearly white papules were present on the prepuce, medial aspect of bilateral thighs, and perianal region (Figures 1, 2). The patient also had multiple, tender, erythematous ulcers on his hard palate, right buccal mucosa, and left angle of mouth (Figure 3). He also had unilateral non-tender enlargement of inguinal lymph nodes.

**Figure 4:**
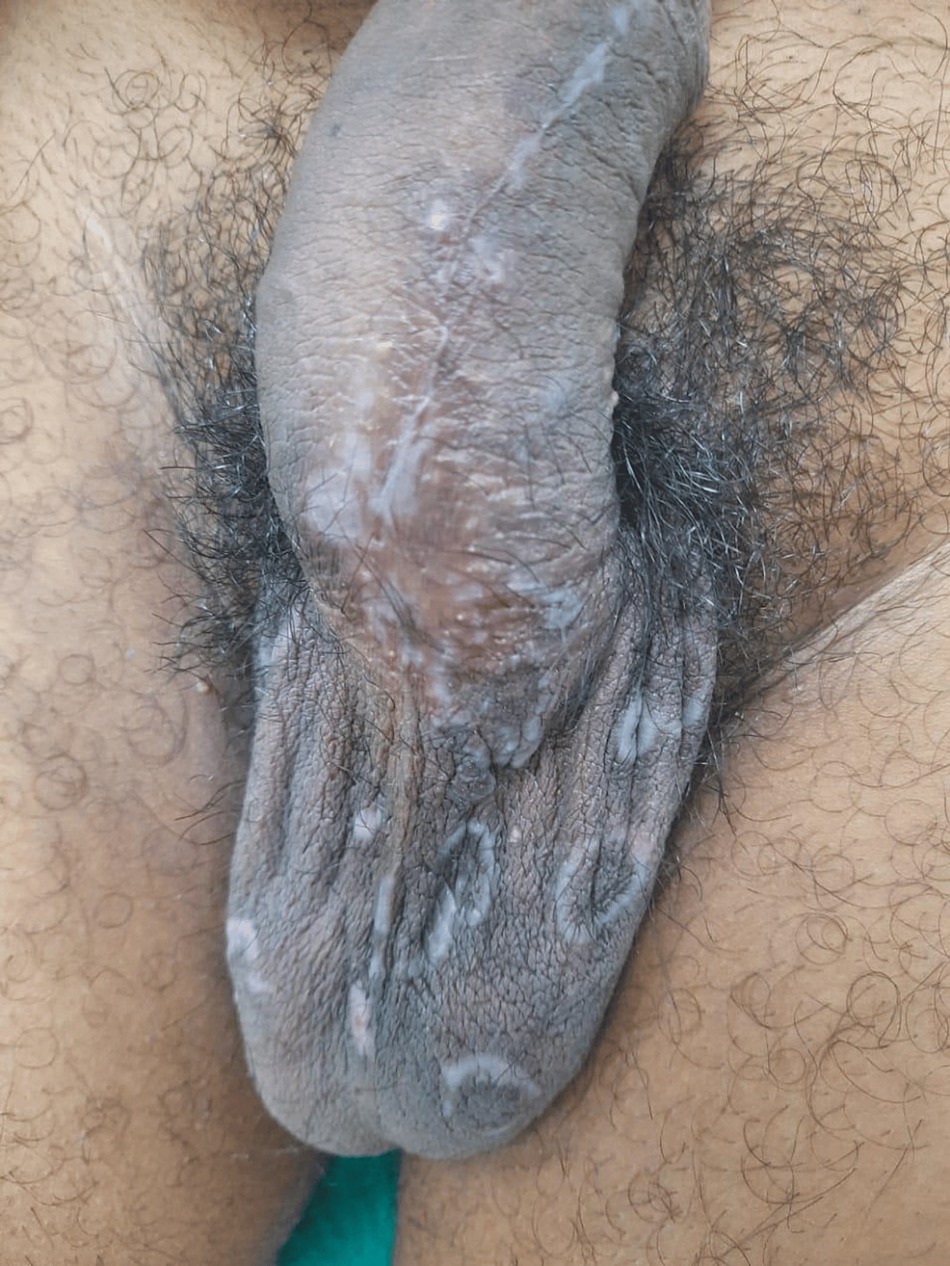
Resolution of lesions post penicillin therapy Immediate regression of the lesions of secondary syphilis, five days after the first dose of benzathine penicillin G

On investigating, the Venereal Disease Research Laboratory (VDRL) test was reactive, with an antibody titer of 1:256. Similarly, Treponema pallidum hemagglutination assay (TPHA) was also positive. A combined serum investigation for HIV by chemiluminescent microparticle immune assay (CMIA) was performed, and the result for anti-HIV-1 antibodies and anti-HIV-2 antibodies was reactive. Only HIV-1 antibodies were detected with immunochromatography. The patient’s CD4+ T lymphocyte count was determined to be 214 cells/µL. Gram stain, Tzanck smear, and herpes simplex virus immunoglobulin G (IgG) and IgM antibodies were negative. The diagnosis of secondary syphilis with concurrent molluscum contagiosum in a laboratory-confirmed HIV patient was established. The patient was initiated on antiretroviral therapy with dolutegravir, lamivudine, and tenofovir disoproxil fumarate, according to the national guidelines for HIV care. The patient was also administered intramuscular benzathine penicillin G 2.4 million units weekly for three consecutive weeks. The umbilicated papules of molluscum contagiosum were treated with weekly cryotherapy sessions. The target lesions resolved completely following the penicillin regimen (Figure 4).

## Discussion

Syphilis can be classified into four phases based on clinical features and laboratory tests: primary, secondary, latent, and tertiary syphilis. A primary lesion arises at the site of spirochete inoculation three to four weeks after incubation, initially presenting as a hard infiltration that progresses into a painless ulcer. These ulcers commonly appear on the genitalia, lips, tongue, buccal mucosa, anus, and fingers.

Secondary syphilis arises from hematogenous dissemination of *T. pallidum*, characterized by severe bacteremia, skin rash, and oral mucosal ulcers. In the early secondary stage, a generalized, non-pruritic papulosquamous rash may develop. Annular, pustular, follicular, vesicular, corymbiform nodular, nodulo-ulcerative (lues maligna), and frambesiform lesions are rare clinical presentations of this stage [6]. Since the onset of the HIV epidemic in the 1980s, there have been numerous isolated reports of coinfection between syphilis and HIV, showcasing uncommon and aggressive manifestations of syphilis specific to each stage of the disease [7].

Individuals with HIV coinfection are more likely to develop multiple and painful chancres in the primary stage. Secondary syphilis symptoms usually arise three to six weeks after the primary chancre resolves; however, there have been numerous reports of overlapping primary and secondary syphilis symptoms in HIV-coinfected patients [8]. In cases of tertiary syphilis and HIV coinfection, atypical presentations may include rapid progression of syphilitic aortitis and neurosyphilis [9,10]. Our patient presented with unusual atypical target-like painful lesions over his genitalia, deviating from the classical presentation.

Studies indicate that patients with painless lesions are more likely to receive presumptive treatment for syphilis [11]. A study involving 446 males with genital ulcers found that clinical differentiation between herpetic ulcers and chancroid was often challenging [12]. Similarly, during a syphilis outbreak in Manchester, numerous cases of multiple, painful ulcers strongly indicative of genital herpes were reported [13]. This highlights a potential diagnostic pitfall, as clinicians may attribute atypical painful genital and extragenital lesions to other causes such as herpes genitalis, chancroid, or neoplasms.

Proper laboratory testing is essential for an accurate diagnosis of syphilis, often referred to as "the great imitator." Two types of serologic tests are used to screen for syphilis, both measuring antibodies in serum or plasma. Non-treponemal assays, such as the rapid plasma reagin (RPR) and Venereal Disease Research Laboratories (VDRL) tests, detect antibodies to lipoidal products like cardiolipin, released from host cell membranes following T. pallidum infection. Treponemal tests like the T. pallidum particle agglutination assay (TP-PA), fluorescent treponemal antibody absorption (FTA-ABS) test, Treponema pallidum haemagglutination assay (TPHA) test measure antibodies specific to T. pallidum proteins. The traditional approach to syphilis serologic screening typically starts with a nontreponemal (lipoidal antigen) test, followed by confirmation of reactive specimens through a treponemal test. The reverse sequence syphilis screening (RSSS) algorithm begins with a treponemal assay as the initial screen, followed by confirmatory testing of reactive samples using a non-treponemal test. Additional confirmatory tests for RSSS include the TPPA test, FTA-ABS, multi-parameter line immunoassays, and Western blots [14]. In our patient, we followed the conventional detection method. Initially, we conducted VDRL in serial dilution to eliminate the prozone phenomenon. Subsequently, confirmation was performed using TPHA. The fourth-generation HIV screening test CMIA was used, as it facilitates early detection of acute HIV infection within a shorter window period. Lateral flow immunochromatography is often utilized for the rapid detection of anti-HIV-1 antibodies and/or anti-HIV-2 antibodies in patients [15]. In our case, immunochromatography detected only anti-HIV-1 antibodies. It has been seen that the atypical manifestations of syphilis in PLHIV are more prevalent in patients with HIV-1 infection [16].

In PLHIV, syphilis infection is said to be associated with a notable rise in HIV viral load and a marked decrease in CD4 cell count. These levels typically revert to pre-syphilis levels or show improvement following syphilis treatment [17].

## Conclusions

In light of our patient's diagnosis of secondary syphilis, concurrent HIV-1 infection, and molluscum contagiosum, prompt interventions were initiated. The administration of benzathine penicillin, antiretroviral therapy, and cryotherapy resulted in successful management and rapid resolution of symptoms.

The increasing prevalence of syphilis, particularly among individuals living with HIV, is a concerning trend. The coexistence of HIV with syphilis has led to a rise in the number of patients presenting with unusual clinical features. Such a scenario poses a diagnostic challenge that leads to inaccurate or delayed treatment. This ultimately contributes to the further spread of the infection.

Our case serves as a reminder for clinicians to maintain vigilance toward the possibility of syphilis infection, even in the absence of the usual clinical manifestations. Timely recognition and accurate management are crucial in halting the disease course and transmission of syphilis. As such, ongoing education and awareness among healthcare professionals are essential to improving diagnostic accuracy and ensuring optimal patient outcomes in the face of evolving infectious disease dynamics.
